# Extracellular Vesicle-Based Detection of Pancreatic Cancer

**DOI:** 10.3389/fcell.2021.697939

**Published:** 2021-07-23

**Authors:** Yesim Verel-Yilmaz, Juan Pablo Fernández, Agnes Schäfer, Sheila Nevermann, Lena Cook, Norman Gercke, Frederik Helmprobst, Christian Jaworek, Elke Pogge von Strandmann, Axel Pagenstecher, Detlef K. Bartsch, Jörg W. Bartsch, Emily P. Slater

**Affiliations:** ^1^Department of Visceral, Thoracic and Vascular Surgery, Philipps University Marburg, Marburg, Germany; ^2^Department of Neurosurgery, Philipps University Marburg, Marburg, Germany; ^3^Department of Neuropathology, Philipps University Marburg, Marburg, Germany; ^4^Core Facility-Mouse Pathology and Electron Microscopy (MPEM), Philipps University Marburg, Marburg, Germany; ^5^Institute for Tumorimmunology, Philipps University Marburg, Marburg, Germany

**Keywords:** pancreatic cancer, extracellular vesicles, ADAM8, serum biomarkers, miRNA

## Abstract

Due to a grim prognosis, there is an urgent need to detect pancreatic ductal adenocarcinoma (PDAC) prior to metastasis. However, reliable diagnostic imaging methods or biomarkers for PDAC or its precursor lesions are still scarce. ADAM8, a metalloprotease-disintegrin, is highly expressed in PDAC tissue and negatively correlates with patient survival. The aim of our study was to determine the ability of ADAM8-positive extracellular vesicles (EVs) and cargo microRNAs (miRNAs) to discriminate precursor lesions or PDAC from healthy controls. In order to investigate enrichment of ADAM8 on EVs, these were isolated from serum of patients with PDAC (*n* = 52), precursor lesions (*n* = 7) and healthy individuals (*n* = 20). Nanoparticle Tracking Analysis and electron microscopy indicated successful preparation of EVs that were analyzed for ADAM8 by FACS. Additionally, EV cargo analyses of miRNAs from the same serum samples revealed the presence of miR-720 and miR-451 by qPCR and was validated in 20 additional PDAC samples. Statistical analyses included Wilcoxon rank test and ROC curves. FACS analysis detected significant enrichment of ADAM8 in EVs from patients with PDAC or precursor lesions compared to healthy individuals (*p* = 0.0005). ADAM8-dependent co-variates, miR-451 and miR-720 were also diagnostic, as patients with PDAC had significantly higher serum levels of miR-451 and lower serum levels of miR-720 than healthy controls and reached high sensitivity and specificity (AUC = 0.93 and 1.00, respectively) to discriminate PDAC from healthy control. Thus, detection of ADAM8-positive EVs and related cargo miR-720 and miR-451 may constitute a specific biomarker set for screening individuals at risk for PDAC.

## Introduction

Pancreatic cancer is the fourth leading cause of cancer-related deaths in the world with an incidence of 45 in 100,000 and a 5-year survival rate of around 9% (Siegel et al., 2020). Among pancreatic cancer, pancreatic ductal adenocarcinoma (PDAC) is the most common type with more than 90% of all cases. A number of factors are responsible for the poor prognosis of PDAC that combines the difficulties in detecting the tumor in early stages, an aggressive biological behavior to account for metastasis and the resistance to existing adjuvant therapies. To detect PDAC in early stages, only a few biomarkers are used routinely that are able to detect its presence and the lesions prior to its derivation (Bartsch et al., 2018). Exosomes are a defined type of extracellular vesicle (EV) ranging in size from 30 to 100 nm and secreted by all cell types including cancer cells. In the context of cancer, exosomes produced in tumor cells contain an abundance of cell-specific molecules that may facilitate the discrimination between cancer afflicted patients and healthy individuals (Sumrin et al., 2018).

Exosome cargo consists of proteins, nucleic acids and lipids (Colombo et al., 2014). Their composition is not only a reflection of the cell they originated from, but also appears to be a regulated process that remains not completely understood (Minciacchi et al., 2015). Content sorting and exosome release seem to not only depend on the type of donor cell, but also on its physiological or pathological state, different stimuli and the pathway of exosome biogenesis (Minciacchi et al., 2015). There are, however, marker proteins that have been found to be specific to EVs because they are related to their pathway of biogenesis. These proteins are, for example, members of the tetraspanin family (CD9, CD81) that are characteristic to exosomes, or flotillins (flotillin-1–2), which are frequently observed in exosomes and microvesicles alike (Kowal et al., 2016). Because exosome content is specific to their donor cell and exosomes can be isolated from bodily fluids such as blood, saliva, ascites or urine, they make promising candidates for early tumor diagnostics.

One rationale to use EVs as early diagnostic markers is their potential to transport tumor-associated micro RNAs (miRNAs) encapsulated in serum exosomes. MiRNAs are small non-coding RNAs of about 18–22 nt long that can be transferred to adjacent cells in the tumor microenvironment to modulate gene expression (Zonari et al., 2013; Su et al., 2016).

As protein cargo, membrane proteins involved in extracellular communication and remodeling are excellent candidates for exosome loading. Among these proteins metalloprotease-disintegrins (ADAM) are potential cargo proteins (reviewed in Shimoda and Khokha, 2017). Due to the interaction with the exosomal marker protein CD9, ADAM10 was one of the first members of the ADAM protease family found to be associated with exosomes (Keller et al., 2009). In previous studies, ADAM8 was defined as an ADAM protease associated with tumor progression and metastasis formation in PDAC (Valkovskaya et al., 2007; Schlomann et al., 2015). In addition, ADAM8 is expressed in tumor associated immune cells such as macrophages, NK cells and neutrophils (Jaworek et al., 2021). These data suggest that ADAM8 itself could be a diffusible molecule that is, similarly to the ADAM protease ADAM10, released in exosomes. Recently, much attention has been addressed to EVs, which may serve as a strategy of monitoring and managing disease status (Cufaro et al., 2019). Since ADAM8 expression is high in PDAC, it is likely that EVs isolated from its precursor lesions, Pancreatic intraepithelial Neoplasia (PanINs) types 2 and 3, could be packed with ADAM8 and ADAM8-associated molecular markers such as miRNAs as shown for breast cancer with correlated expression levels of ADAM8 and miRNA-720 (Das et al., 2016). MiRNAs are integral components of almost every cancer-related biological process, including cellular differentiation, proliferation, migration, apoptosis, EMT and angiogenesis. Here we hypothesized that high ADAM8 expression levels in pancreatic cancer is reflected by release of EVs expressing ADAM8 on their surface and that ADAM8 expression might cause miRNAs associated with ADAM8 expression to be cargo for ADAM8-positive EVs, so that these EVs can be used to detect pancreatic cancer in patient serum at early stages.

## Materials and Methods

### Patient Cohort

Patients with familial pancreatic cancer (FPC) or PDAC treated at the Department of Visceral Surgery at University Hospital Marburg were enrolled in our study. All patients provided written informed consent prior to participating in this study. Ethical approval was granted from the local ethics committee at Marburg University, Faculty of Medicine (File No. 5/03). All tumors were histologically staged by an experienced pathologist according to the UICC-TNM (Union for International Cancer Control; tumor, node, metastasis) classification 2017 (Gospodarowicz and Brierley, 2017).

### Extracellular Vesicle Preparation

250 μl of serum were diluted with 4.5 ml Hank’s Salt Saline Buffer in a 15 ml falcon tube in order to lower sample viscosity and centrifuged at 800 × g for 5 min in order to eliminate any remaining cells. The supernatant was transferred to a new 15 ml falcon tube and centrifuged at 2,000 × g for 10 min to remove dead cells or cell debris (Théry et al., 2006; Melo et al., 2015). The supernatant was then transferred to a 5 ml syringe and filtered through a 0.2 μm pore filter and transferred to a 6.0 ml polypropylene bell-top quick-seal centrifuge tube and filled with Hank’s Balanced Salt Solution (HBSS). The tubes were centrifuged at 100,000 × g and 4°C for 70 min. The pellet was resuspended in HBSS and transferred to a polypropylene 1.5 ml microcentrifuge tube and was centrifuged with an Optima MAX-XP Ultracentrifuge in a TLA-55 fixed angle rotor at 100,000 × g and 4°C for 100 min. The supernatant was discarded, and the pellet resuspended with 50 μl HBSS. Samples were stored at –80°C. In order to determine the particle size and concentration of isolated EVs, NTA was performed with the ZetaView^®^ BASIC PMX-120 and the corresponding software ZetaView^®^.

### Western Blot Analysis

To further phenotype isolated EVs, enriched proteins were detected in Western blots. Twenty μg protein determined by standard BCA were boiled in Laemmli buffer without β-Mercaptoethanol (60 mM Tris-HCl, pH = 6.8; 2% SDS; 10% Glycerol; 0.01% Bromphenol-Blue) for 5 min. Protein separation was performed by SDS-PAGE followed by a transfer onto PVDF membranes. Successful transfer was confirmed by Ponceau S staining. To block unspecific binding, membranes were immersed in 4% BSA in TBST (50 mM Tris, pH = 7.5; 150 mM NaCl; 0.1% Tween-20) for 1 h, followed by incubation with primary antibody against CD9 (CBL162; Chemicon International, Temecula, CA, United States, 1:1,000 in 4% BSA in TBST) at 4°C overnight. After washing three times, blots were incubated with the respective secondary antibody for 1 h. After an additional washing step, signals were detected with SuperSignal^TM^ West Pico PLUS Chemiluminescent Substrate (Thermo Fisher Scientific, Rockford, IL, United States). Additional antibodies were used to characterize the EVs isolated. These included CD81 (sc-166029; Santa Cruz Biotechnology), Flotillin-1 (PA5-18053; Thermo Fisher Scientific), hADAM8 (MAB10311; R&D Systems) and Calnexin (2679; Cell Signaling Technology).

### FACS Analyses

FACS analyses were performed as previously described (Bartsch et al., 2018). Briefly, 1.5 × 10^9^ EVs were coupled to 10 μl of 4 μm aldehyde/sulfate latex beads, 4% w/v in 100 μl PBS and incubated for 15 min at room temperature. Then 900 μl of PBS were added to reach a final volume of 1,000 μl and EVs and beads were incubated for another 30 min at room temperature. Beads were then blocked by adding 50 μl 10% BSA. Samples were centrifuged at 9,900 rpm for 1 min. Supernatant was discarded leaving a volume of 100 μl in the tubes. Samples were further blocked by incubation with 5 μl Human True Stain FcX Blocking Solution for 10 min at room temperature. Samples were then centrifuged again at 9,000 rpm for 1 min and the supernatant was discarded. Pellets were resuspended in 20 μl 2% BSA. Now, one half of the samples was incubated with 3 μl anti-ADAM8 (MAB10311, R&D Systems) at 4°C overnight. The next day, all samples were washed in 1 ml 2% BSA and centrifuged at 9,900 rpm for 1 min. All samples were treated with 20 μl of a blocking solution consisting of Human True Stain FcX Blocking Solution (BioLegend, San Diego, CA, United States) and 2% BSA and incubated with 3 μl alexa-488-tagged secondary antibody (Abcam, Cambridge, United Kingdom) at 4°C for 1 h. In a final step, samples were washed twice in 1 ml 2% BSA. The final bead pellet was resuspended in 1 ml PBS and transferred to 5 ml Flow Cytometry Tubes. Samples were stored at 4°C in the dark until measurement. The percentage of ADAM8-positive beads out of 100,000 total events was then calculated in a FACS analysis.

### Electron Microscopy

EVs were stained for electron microscopy as previously described (Théry et al., 2006). Briefly, purified extracellular vesicles were fixed with an equal amount of 4% PFA. An amount of 5–7 μl was placed on a Formvar/carbon coated 200 mesh copper (Ted Pella Inc., Redding, CA) electron microscopy grid and incubated for 20 min. After the membrane adsorbed the vesicles the grids were washed with sterile filtered PBS and fixed for 5 min with 1% glutaraldehyde. The grids were washed 8 times for 2 min with sterile filtered water and then incubated with 1% uranyl acetate for 5 min. After an additional incubation with 2% methyl cellulose supplemented with 4% uranyl acetate (ratio 9:1) on ice, the excess fluid was removed with filter paper and the grids were air dried for up to 10 min. The exosomes were imaged with a Zeiss EM 900 at 80 kV.

### Protease Activity Assay

Serum EVs isolated from either PDAC patients or healthy individuals were tested for ADAM8 activity by determining cleavage of a FRET-based polypeptide substrate with a high Kcat/Km for ADAM8 (PEPDab13, BioZyme, Inc., North Carolina, United States) as previously described (Schlomann et al., 2019). Briefly, 10 μM of PEPDab13 in 50 μl assay buffer (1 mM ZnCl_2_, 20 mM Tris-HCl pH 8.0, 10 mM CaCl_2_, 150 mM NaCl, 0.0006% Brij-35) was incubated with 3.75 × 10^8^ EVs in a total volume of 100 μl. Resulting fluorescence was monitored every 2 min for 6 h at 37°C with a multiwell plate reader (FLUOstar OPTIMA, BMG Labtech, Offenburg, Germany) using λ_*ex*_ of 485 nm and an λ_*em*_ of 530 nm.

### Serum Exosome miRNA Analysis

Total RNA carried by exosomes or other EVs in 250 μl of serum was extracted using the ExoRNeasy Serum/Plasma Midi Kit from Qiagen (Hilden, Germany) with the addition of a spike of 25 fmol of synthetic cel-miR-54 DNA as recommended by the manufacturer. The RNA was converted to cDNA using the miRNA Reverse Transcription Kit, miScript II RT Kit, also from Qiagen. The cDNA synthesis reaction was diluted and incubated with QuantiTect^®^ SYBR Green PCR Master Mix, miScript Universal Primer and specific miScript Primer Assays. The real-time PCR reactions were run in a StepOnePlus Real time PCR System from Applied Biosystems (Darmstadt, Germany). The Δ threshold cycle (Ct) values were then calculated by subtracting the *cel*-miR-54 Ct value from the specific miRNA Ct value. For the analyses of cell lines SNORD95 was chosen as the endogenous control as previously described (Sperveslage et al., 2014). Ct values of each target miRNA transcript were normalized against the Ct value of SNORD95. Relative change in exosomal miRNA expression comparing wild type and knock-out cells was calculated using the ΔΔCt method.

### Statistical Analyses

A Wilcoxon signed-rank test and a *t*-test were performed to assess whether the patient values were significantly different from control samples. A *p*-value of < 0.05 was considered to be statistically significant. The receiver operating characteristic curve analyses were performed using GraphPad Prism version 6 (GraphPad Software, La Jolla, CA, United States).

## Results

### Clinicopathological Characteristics of the Recruited Patients, Including IAR With High-Risk Precursor Lesions

The characteristics of the 72 PDAC patients that were included in the study are presented in Table 1.

**TABLE 1:** Clinicopathological characteristics of the recruited patients.

		**Cohort (*n* = 72)**
**Gender**		
	Males (%)	37 (51%)
	Females (%)	35 (49%)
Median age at surgery, years (range)		68 (47–85)
**UICC stage**		
	I	11 (15.3%)
	II	10 (13.9%)
	III	46 (63.9%)
	IV	5 (6.9%)
Median survival, months (range)		22 (1–92)
**Location**	Pancreas	
	Head	65 (90.3%)
	body or tail	7 (9.7%)

*In addition, 7 individuals at risk (IAR) who had undergone surgery for removal of precursor lesions were also recruited. These included 2 males and 5 females with a median age of 54 years. Histologically verified precursor lesions included 1 main duct intraductal papillary mucinous neoplasm (IPMN) with high grade dysplasia, 2 PanIN 3 and 4 PanIN 2. The healthy controls (n = 20) had a median age of 40 years and included 10 males and 10 females.*

### Preparation of Extracellular Vesicles From Patient Serum Samples

**FIGURE 1:**
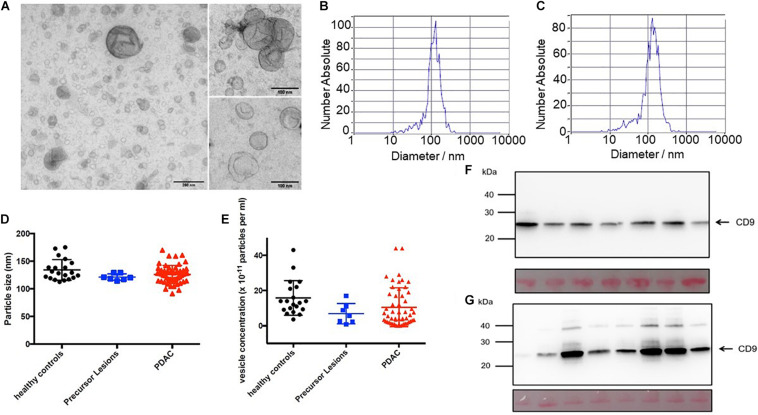
Extracellular vesicles prepared from serum. Electron micrograph of EVs isolated from PDAC patient’s serum **(A)**. Border is clearly delimited. ZetaView measurements of serum EVs isolated from healthy control **(B)** and PDAC patient **(C)**. Mean particle size **(D)** and mean vesicle concentration **(E)** of serum EVs from 7 patients with pancreatic precursor lesions (blue) and 52 patients with PDAC (red) compared to 20 healthy individuals (black). Immunoblots of serum EVs isolated from healthy individuals (*n* = 7, **F**) and PDAC patients (*n* = 8, **G**). Twenty μg protein determined by standard BCA were separated in an SDS-PAGE, blotted to PVDF membranes, stained with Ponceau S to confirm equal loading (lanes below), and probed with antibody against exosomal protein CD9.

Extracellular vesicles including exosomes with an average diameter < 120 nm were isolated from patient serum using a range of purification steps including ultracentrifugation and filtration. The exosomes present in these preparations were identified by a number of analytical methods including ZetaView^®^ analyses and electron microscopy to confirm existence of a double membrane and the proper size corresponding to exosomes (Figures 1A–C). The particle size and vesicle concentration did not vary among the preparations from the different sources (Figures 1D,E). In addition, CD9 was found in all preparations. However, whereas the control samples had relatively constant amounts of CD9 (Figure 1F), the tumor samples varied in abundance (Figure 1G). Additional markers were also tested to characterize the EVs. The preparations were also positive for CD81, Flotillin-1 and ADAM8, but were negative for calnexin (Supplementary Figure 1).

### Diagnostic FACS Analysis of ADAM8 in Exosomes

**FIGURE 2:**
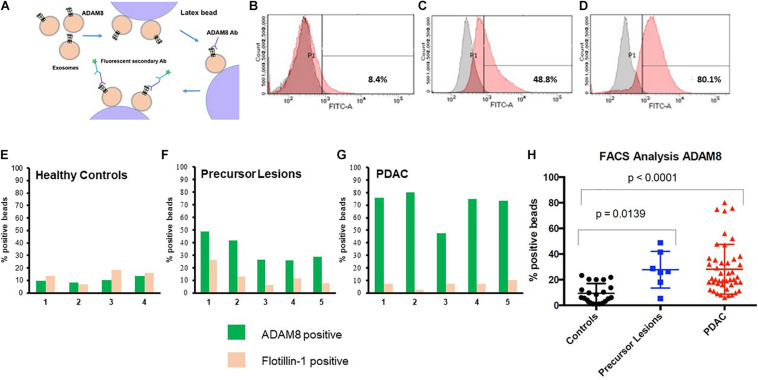
Cartoon of protocol for flow cytometry (FACS) **(A)**. FACS analyses of serum EVs isolated from healthy individuals **(B)**, patients with precursor lesions **(C)** or PDAC **(D)**, attached to beads and detected with ADAM8 antibody. Results with secondary antibody alone appear in gray and those with both ADAM8 antibody and secondary antibody appear in red. Exemplary comparison of results for FACS analyses of serum exosomes from healthy controls (*n* = 4, **E**), precursor lesions (*n* = 5, **F**) or PDAC (*n* = 5, **G**) with ADAM8 (green) or Flotillin-1 (ochre) antibodies. Graphic representation of results from FACS analysis of EVs isolated from healthy individuals, patients with precursor lesions or PDAC from the total cohort, attached to beads and detected with ADAM8 antibody **(H)**.

In order to detect ADAM8 on the surface of exosomes, a bead-coupled FACS analysis was performed (Figure 2). Positive ADAM8 signals were observed both in control individuals and in PDAC patients. However, their proportion was significantly different, so that an enrichment of ADAM8 in serum exosomes from patients with PDAC or its precursor lesions compared to healthy individuals was observed (*p* < 0.0001 or *p* = 0.0139, respectively).

### Cargo Analysis of Serum Derived EVs From Control and PDAC Patients

**FIGURE 3:**
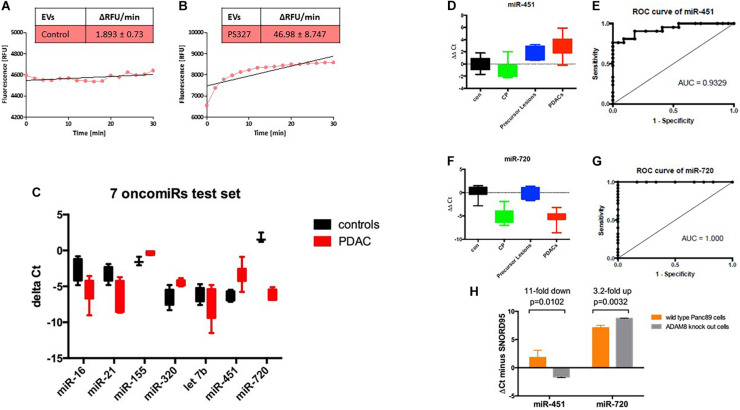
Activity assay of ADAM8 in serum EVs isolated from healthy control **(A)** or PDAC patient **(B)**. Results of the test set using 7 oncomiRs with black boxes, controls, and red boxes, PDAC patients **(C)**. Boxplots of the (miR-451–*Cel*-54) **(D)** and (miR-720—*Cel*-54) **(F)** ΔΔCt values (patient minus healthy control) in the four groups (con, healthy control; CP, chronic pancreatitis; precursor lesions; and PDACs, pancreatic ductal adenocarcinoma). ROC curves for the comparison of exo-miR-451 **(E)** and exo-miR-720 **(G)** expression levels between patients with PDAC and healthy controls. ΔCt values for miRNAs isolated from EVs secreted by wild type Panc89 (orange) and ADAM8 knock-out cells (gray) **(H)**.

Since ADAM8 is located on EVs as shown by bead-coupled FACS analysis, we investigated whether ADAM8 confers enzymatic activity to EVs enriched in ADAM8. Activity of ADAM8 can be detected by using a FRET-based peptide representing the cleavage site of CD23 (Schlomann et al., 2019). Cleavage analysis of EVs isolated from either a control individual or a PDAC patient revealed a strongly enhanced proteolytic activity in EVs from the PDAC patient (Figures 3A,B). Although the FRET-based peptide is not specific for ADAM8 activity, it is very likely that the increased activity originates from ADAM8 as a protease with enhanced expression in EVs from PDAC patients as the FACS analysis suggests. In addition to the protein cargo analysis, a systematic screening for miRNAs was performed on EVs derived from control individuals and PDAC patients (Figure 3C). Interestingly, a set of 7 oncomiRNAs were found to be differentially regulated with the strongest upregulation for miRNA-451 and the strongest down-regulation for miRNA-720 (Figure 3C). To confirm differential regulation of these miRNAs in the PDAC patient cohort, exosomal miRNAs were isolated from the same serum samples and analyzed for miR-720 and miR-451 by semi-quantitative real time RT-PCR. Serum samples had been spiked with synthetic *C. elegans* miR-54 before miRNA isolation to be used as a normalization control. Statistical analyses were performed using the Wilcoxon rank test and ROC curve analysis. The miR-720 and miR-451 were also diagnostic, as patients with PDAC had significantly higher serum exosome levels of miR-451 and lower serum exosome levels of miR-720 than healthy controls and reached high sensitivity and specificity with an AUC = 0.9329 and 1.000, respectively, to discriminate PDAC (Figures 3D–G). In addition, serum exosomes were also isolated from patients with chronic pancreatitis (CP; *n* = 10) and precursor lesions (*n* = 7). Whereas serum exosomes from patients with precursor lesions had increased levels of miR-451, approaching the levels found in PDACs, CP patient serum exosomes did not. In contrast, the serum exosomes isolated from CP patients had lower levels of miR-720, similar to the PDAC serum exosomes, while the exosomes derived from serum of patients with precursor lesions had levels comparable to the healthy controls. Analysis of these miRNAs in Panc89 wild type and ADAM8 knock-out cells demonstrated that the levels of exosomal miR-451 decrease and the levels of exosomal miR-720 increase upon knock-out of ADAM8, suggesting a regulatory component of ADAM8 on these miRNAs (Figure 3H).

## Discussion

EV based serum diagnostics provides an additional and powerful diagnostic component in the field of liquid biopsies (Yee et al., 2020). With regard to EV diagnostic in PDAC, the concentration and size of EVs in patient serum has been correlated with tumor differentiation and overall survival in PDAC patients (Badovinac et al., 2021), but no specific cargo analysis of “diagnostic” EVs in serum has been reported up to now. In this respect, our results provide some novelties: First of all, we identified ADAM8, a protease with a therapeutic potential in PDAC, to be located in EVs that meet all criteria for exosomes. By establishing a bead-supported FACS analysis method to analyze surface located ADAM8 in EVs, we demonstrated that ADAM8-positive EVs were significantly enriched in PDAC patients and were gradually increased with increasing tumor staging, at least when comparing precursor lesions with fully developed adenocarcinoma. From the biochemical point of view, we hypothesized that ADAM8 integrated in EV membranes should be enzymatically active. By peptide cleavage assays, we were able to confirm that ADAM8-enriched EVs show remarkable activities compared to those EVs from control individuals. These data support the notion that a FACS based analysis of EVs from PDAC patients can be performed to detect membrane proteins that are topologically oriented to the extracellular compartment.

In addition to the pure presence of ADAM8, we investigated potential exosomal miRNAs as EV cargo that could be regulated by ADAM8. A panel of “oncomiRNAs” including let-7b was screened from exosomal miRNAs extracted from healthy control individuals and PDAC patients, respectively. We found that ADAM8-positive EVs are specifically equipped with miRNAs that show a functional relevance in PDAC as exemplified by the results with miR-451 and miR-720 in serum EVs from these patients. From all miRNAs examined, exosomal (exo)-miRNA-720 and exo-miRNA-451 were the most significantly dysregulated. Exosomal miRNA-720 was significantly down-regulated in serum samples from chronic pancreatitis and PDAC patients and therefore suggested perfect accuracy in the diagnosis of CP and PDAC either in its hereditary or sporadic form (AUC = 1), exosomal miRNA-451 showed the highest up-regulation in precursor lesions and in PDAC, but not in samples from CP patients and was able to discriminate between precursor lesion or PDAC-afflicted patients and healthy individuals with relatively high accuracy and an AUC of 0.9329.

To further analyze the correlation of ADAM8 expression with miRNA expression levels, we used the PDAC cell line Panc89 with a genetic knockout of the *ADAM8* gene (Cook et al., manuscript in preparation). Using these cell lines, we further demonstrate that the regulation of miRNA-451 and miRNA-720 is dependent on ADAM8 expression levels, respectively. ADAM8 expression is inversely correlated with miRNA-720 levels, as an ADAM8-knockout in Panc89 cells leads to an increase in miRNA-720 levels (average 3.2-fold higher in Panc89_A8KO cells vs. Panc89_A8Ctrl cells), in accordance with the finding that we found decreased levels of miRNA-720 and increased ADAM8 levels in PDAC patients compared to control individuals. In contrast, miRNA-451 is positively correlated with ADAM8 expression levels, as this miRNA is decreased in Panc89_A8KO cells vs. Panc89_A8Ctrl cells. Similarly, we found increased miRNA-451 levels in PDAC patients with a higher ADAM8 expression. For both these miRNAs, functional roles in PDAC were reported that are in accordance with their described abundance in PDAC patient sera. In particular, it was shown that miRNA-720 inhibits pancreatic cancer cell proliferation and invasion by directly targeting cyclin D1 (Zhang et al., 2017) so that down-regulation of miRNA-720 as observed in PDAC patients compared to healthy individuals has a potential tumor-promoting effect. ADAM8-dependent regulation of miRNA-720 was reported earlier in the breast cancer cell line MDA-MB-231 (Das et al., 2016) however in the opposite direction, an observation that we could reproduce with a CRISPR/Cas9 generated knockout of *ADAM8* in this cell line. In contrast, miRNA-451 can promote cell proliferation and metastasis in PDAC by down-regulating CAB39 (Calcium binding protein 39), a tumor suppressor upstream of STK11 (serine-threonine kinase 11) (Guo et al., 2017). Thus, as observed here, upregulation of miRNA-451 downregulated a tumor suppressor pathway. With miRNA-451 detecting precursor lesions and PDAC, but not chronic pancreatitis (CP) and mi-RNA 720 detecting CP but not precursor lesions in conjunction with ADAM8-positive EVs, we can achieve a high degree of specificity and sensitivity in serum EV analysis to predict pancreatic precursor lesion and PDAC while discriminating between chronic pancreatitis patients and healthy individuals.

## Conclusion

Enrichment of ADAM8 in serum exosomes as well as the measurement of exosomal miRNAs, miR-720 and miR-451, may contribute to a biomarker profile for the screening of individuals for PDAC. More generally, our data provide evidence for an EV based communication in the PDAC tumor microenvironment that can be triggered in the pro-oncogenic direction by the presence of ADAM8. This biological signature in turn can be exploited for diagnostic purposes to detect PDAC lesions and fully developed PDAC, as demonstrated here.

## Data Availability Statement

The raw data supporting the conclusions of this article will be made available by the authors, without undue reservation.

## Ethics Statement

The studies involving human participants were reviewed and approved by the Ethics Committee of the Marburg University, Faculty of Medicine (File No. 5/03). The patients/participants provided their written informed consent to participate in this study.

## Author Contributions

ES, DB, and JB conceived the study. YV-Y, JF, AS, SN, LC, NG, FH, CJ, EP, and AP performed the experiments and provided resources. ES and JB wrote the manuscript. All authors approved the submitted version.

## Conflict of Interest

The authors declare that the research was conducted in the absence of any commercial or financial relationships that could be construed as a potential conflict of interest.

## Publisher’s Note

All claims expressed in this article are solely those of the authors and do not necessarily represent those of their affiliated organizations, or those of the publisher, the editors and the reviewers. Any product that may be evaluated in this article, or claim that may be made by its manufacturer, is not guaranteed or endorsed by the publisher.
