# Supplementary material for: Extracellular Vesicle-Based Detection of Pancreatic Cancer
Source: Front Cell Dev Biol. 2021 Jul 23;9:697939. doi: 10.3389/fcell.2021.697939 (PMC8343017; doi:10.3389/fcell.2021.697939)
Supplement: Supplementary file 1 [file Data_Sheet_1.PDF]

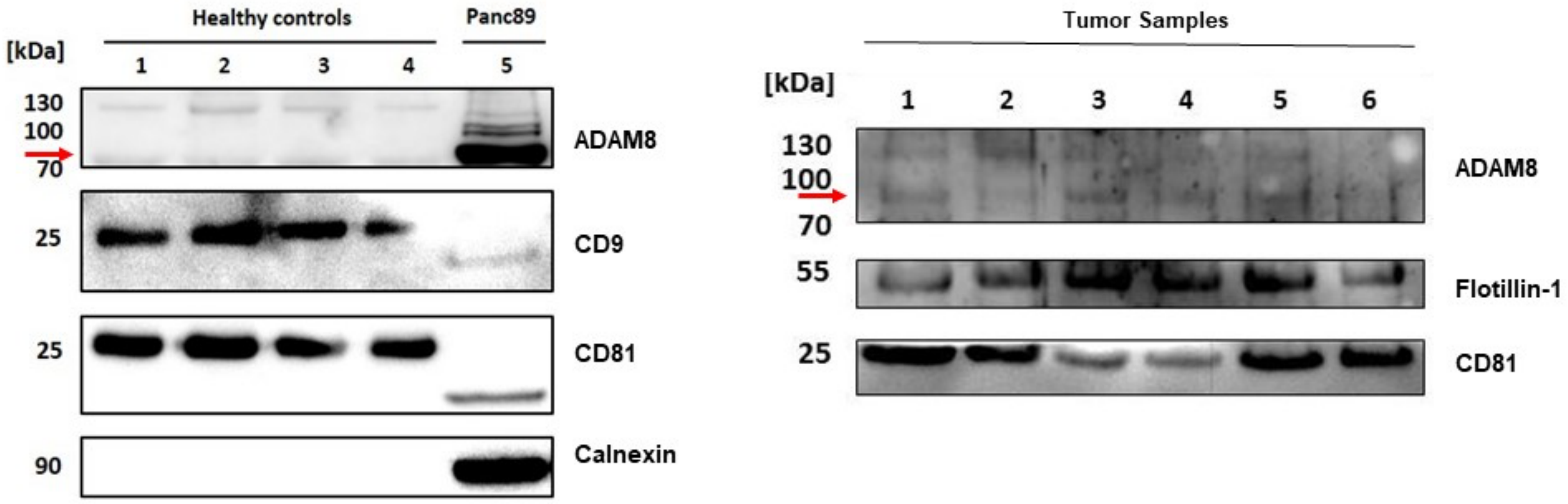

Figure S1. Western blot analysis of our EVs demonstrate the presence of CD9, ADAM8, Flotillin-1 and CD81 and the absence of Calnexin. ADAM8 is only detected in EVs derived from serum of tumor patients (upper right panel) and in the Panc89 cell line (left panel, lane 5), designated by the red arrow, but not in healthy controls (upper left panel).
